# Surface Modification of Poly(lactic acid) Film via Cold Plasma Assisted Grafting of Fumaric and Ascorbic Acid

**DOI:** 10.3390/polym13213717

**Published:** 2021-10-28

**Authors:** Asma Abdulkareem, Peter Kasak, Mohammed G. Nassr, Abdelrahman A. Mahmoud, Mahmoud Khatib A. A. Al-Ruweidi, Khalid J. Mohamoud, Mohammed K. Hussein, Anton Popelka

**Affiliations:** 1 Center for Advanced Materials, Qatar University, Doha P.O. Box 2713, Qatar; asma.alkareem@qu.edu.qa (A.A.); mn1402336@student.qu.edu.qa (M.G.N.); am1306019@qu.edu.qa (A.A.M.); ma1207471@qu.edu.qa (M.K.A.A.A.-R.); km1306840@student.qu.edu.qa (K.J.M.); mh1300782@student.qu.edu.qa (M.K.H.); 2 Department of Chemistry and Earth Sciences, College of Arts & Sciences (CAS) Qatar University, Doha P.O. Box 2713, Qatar

**Keywords:** poly(lactic acid), PLA, ascorbic acid, fumaric acid, plasma treatment, grafting, wettability, adhesion

## Abstract

Plant-based materials have found their application in the packaging with a yearly growing production rate. These naturally biodegradable polymers are obtained from renewable and sustainable natural resources with reduced environmental impact and affordable cost. These materials have found their utilization in fully-renewable plant-based packaging products, such as Tetra Pak^®^-like containers, by replacing commonly-used polyethylene as the polymer component. Poly(lactic acid) (PLA) is one of the representative plant-based polymers because of its eco-friendliness and excellent chemical and mechanical properties. In this work, a PLA surface was modified by various food additives, namely ascorbic acid (ASA) and fumaric acid (FA), using plasma-initiated grafting reactions in order to improve the surface and adhesion properties of PLA. Various analytical and microscopic techniques were employed to prove the grafting process. Moreover, the improved adhesion of the modified PLA foil to aluminum (Al) foil in a laminate configuration was proven by peel resistance measurements. The peel resistance of modified PLA increased by 74% and 184% for samples modified by ASA and FA, respectively, compared with untreated PLA.

## 1. Introduction

Global environmental policy and market knowledge of the use and consumption of materials with low environmental effects are the first guiding factors in the production and investigation of new biodegradable or organic products. The packaging industry is one of the most critical waste generators [1]. Current consumption patterns in our society are producing a great deal of waste that needs to be carefully handled to generate the least environmental effects. Plastic, apparently, constitutes a significant part of this form of waste, and it is challenging and costly to recycle due to its petrochemical origin [2], primarily due to the current problem of separation. Accordingly, polymeric materials occupy core study lines because of the importance that polymers obtained from sustainable sources (plant-based polymers) substitute petroleum-based polymers [3,4,5,6,7]. Poly(lactic acid) (PLA) is currently the most promising biodegradable, compostable and renewable polymer in synthetic form that can be completely degraded [8,9]. PLA is a linear thermoplastic aliphatic polyester that can be produced by the ring opening polymerization of lactide. Lactide, as a cyclic dimer, is made from the controlled depolymerization of lactic acid oligomers, which is are obtained from sugar feedstock fermentation, corn fermentation, and other sources [10,11,12]. PLA has excellent mechanical properties, high stiffness, biodegradability [13], biocompatibility [13], bio-absorbency [13], transparency [14], gas permeability [14], low toxicity [15], ease of processing [15], and UV resistance [15]. Due to these facts, PLA also has found a wide range of applications such as in the agricultural, biomedical, and packaging industries (specifically food packaging applications) [16,17,18]. Nevertheless, neat PLA possesses low surface free energy (wettability) resulting in weak adhesion and thus, limits the wider usage of PLA in the packaging industry. In recent years, many research studies have been reported about improving PLA properties by fusion with different additives such as lubricants, plasticizers or other polymers [19,20,21]. However, this approach is not recommended for food packaging applications because it can generate waste that may have the potential to harm the environment and health [3]. Consequently, treatments that overcome such issue waste are of increasingly high importance. As a response to this issue, surface modification is a safe, simple, and affordable option [22,23,24]. Surface modification methods can be physical or chemical, and there are a variety of treatments available. Over the last few decades, these modification methods have been established with an emphasis on the surface incorporation of extra functionalities responsible for the growing increase of surface free energy (wettability) and tailored applications [25,26,27]. Such modifications should only affect the top surface area (tens of nm), which is essential for functional strength and at the same time to maintain integrity of the material. For this reason, plasma technology has become the preferable choice as a representative technique used for physical treatment. As the fourth state of matter, plasma is an ionized gas representing a mixture of electrons, ions, metastatic ions, and free radicals with enough energy to modify a treated surface [23,28,29,30]. During plasma treatment, the activated species reach the surface of the substrate and break up the molecular chain, forming new functional groups (mostly containing oxygen). Furthermore, surface activation can be induced by atom substitution and/or recombination, which is the most important mechanism [31]. This leads to improved roughness, adhesion, wettability and protection of material [32]. In food packaging applications, antibacterial packaging materials are used to keep food from being vulnerable to microorganisms and to extend the shelf life of food [33,34,35]. To create biomaterials, some antibacterial procedures include combining antibacterial agents inside the polymeric content [36,37]. Nevertheless, this method is not ideal for all packaging materials because it alters the materials’ key physical and mechanical properties and reduces their stability. Therefore, the development of PLA based films with enhanced antimicrobial properties is highly demanded. For this purpose, the PLA surface can be modified with antimicrobial agents using the plasma technique as an effective radical initiator for the grafting processes [38]. Due to their nontoxicity, biocompatibility, and antimicrobial properties, ascorbic acid (ASA) and fumaric acid (FA) are promising materials for this modification [39,40]. These material compounds are components of organic biosynthesis in humans, essential bioactive species, and conservant, and they are often used in medicinal applications [41]. Therefore, for enhancement of the adhesion of PLA to Al as a component towards packaging applications, a rapid fabrication process is required combining cold plasma treatment as a simple, scalable and physical surface modification technique together with a surface modification by addition of cost effective, sustainable and biocompatible molecules.

Thus, in this research study, commonly used food additives such as ASA and FA were grafted on the surface of PLA film using low-temperature plasma acting as an initiator of radical grafting reactions. Furthermore, the effect of non-thermal plasma treatment and grafting on the adhesion improvement of PLA film was investigated.

## 2. Materials and Methods

### 2.1. Materials

Commercial poly(lactic acid) (PLA) with a D-isomer content of 4.3%, Mw of 2.53 × 10^5^ g/mol, melt flow index of 6 g/10 min (190 °C/2.16 kg), and density of 1.24 g/cm^3^ was supplied in pellets from (NatureWorks, Minnetonka, MN, USA), which was used for the PLA film preparation. Ethylene glycol (>98% FLUKA, Overijse, Belgium), ultra-pure water (prepared by Purification System Direct Q3, Rue de Luzais, France), and formamide (>98% FLUKA, Overijse, Belgium) were utilized as testing liquids to study wettability. Aluminum foil (Al) (GLAD, Qingdao, Shandong, China) was used to prepare the adhesive joint with PLA. Fumaric acid (FA) C_4_H_4_O_4_, with an average Mw of 116.07 g/mol was purchased from Merck KGaA, (Darmstadt, Germany) and L-ascorbic acid (>99.0% Research-Lab, Uran Islampur, India), molecular weight = 176.14 g/mol and sodium iodide: extra pure, MW 197.89 g/mol purchases from Research-Lab, Uran Islampur (India) were used for the PLA modification. Glacial acetic acid (CH_3_COOH) supplied from VWR International (BDH) CHEMICALS (Radnor, PA, USA), anhydrous (≥99.9% purity) and sodium thiosulfate pentahydrate Na_2_S_2_O_3_.5H_2_O: extra pure (crystals), MW 248.17 g/mol, purchased from Research-lab Fine Chem Industries (Mumbai, India) were used for hydroperoxides determination. All the chemicals were used without further treatment.

### 2.2. Preparation of PLA Films and PLA/Al Laminate

A hot mounting press machine AutoFour/3012-PL,H (Carver, Wabash, IN, USA)was used to fabricate thin, homogeneous PLA films with a thickness of around 0.3 mm. The PLA pellets were melted and pressed with a force of two tones for two minutes at 180 °C in order to achieve a smooth surface in the film. Then the PLA samples were cooled down using a water medium to room temperature and subsequently were washed thoroughly with ethanol to remove any chemicals, residuals, or possible contaminants from the molding process that could affect the surface properties, and dried for 20 min at room temperature under ambient air. Finally, the film samples were sliced into narrow strips (5 cm × 1 cm) and used directly for surface treatment and analysis. Furthermore, 10 cm × 10 cm samples were placed between two steel plates covered by Al foil and molded into a thin film at 160 °C via the hot mounting press machine AutoFour/3012-PL,H (Carver, Wabash, IN, USA) for 2 min at a force of 1 ton. The film was allowed to cool down until it reached 30 °C and was then cut into 2 × 10 cm strips in order to be used for a peel test.

### 2.3. Surface Modification of PLA Using Plasma Treatment

PLA films were treated with a low-temperature plasma at vacuum pressure using the RF plasma equipment Venus75-HF (Plasma Etch Inc, Carson, CA, USA). The plasma discharge was generated at radiofrequency (RF) powered electrodes with a capacitive parallel plate design operating at a frequency of 13.56 MHz. A cylindrical chamber made of an Al chamber containing PLA samples was vacuumed to 0.2 Torr, and the plasma treatment was applied for 30 to 180 s at 80 W nominal power. The PLA samples were treated from both sides.

### 2.4. Grafting Process

The prepared PLA films were subsequently modified by an antimicrobial agent using plasma treatment as an initiator for a radical grafting mechanism. Then, the antimicrobial agents ASA and FA were grafted to the surface of the prepared PLA films using low-temperature plasma as initiator for radical grafting. Grafting was carried out for 24 h on plasma-treated PLA samples immersed in ASA 10% *w/v* aqueous solution and in FA 5% *w/v* ethanol solution. Subsequently, the samples were thoroughly washed and dried. The graft yield was obtained gravimetrically to prove the grafting of ASA and FA on the PLA surface using three different samples. The graft yield (GY) was calculated by Equation (1):

GY [%] = ((W_2_ − W_1_)/W_1_) × 100% (1)

where, W_1_ and W_2_ represent the weights of the PLA samples before and after the modification, respectively.

### 2.5. Wettability Analysis

Static contact angle measurements were used to assess the changes in wettability of the plasma-treated and grafting modified PLA surface. For this study, an OCA35 optical contact-angle measurement device (DataPhysics, Filderstadt, Germany) was used, which was equipped with a high-resolution CCD camera. As testing liquids, ultra-pure water, ethylene glycol, and formamide were used. To eliminate the gravitational effect, a 3 µL droplet was dispensed on the sample in ambient air. The contact angle was recorded approximately after 3 s (attainment of thermodynamic consistency between the sample interfaces and the liquid). The average value of testing contact angle value was calculated using five independent measurements in various positions. The total surface-free energy, as well as its polar and dispersive components, were calculated using the Owens, Wendt, Rabel and Kaelble model.

### 2.6. Hydroperoxides Determination

To detect hydroperoxides created on the PLA surface after plasma treatment, a modified iodometric method based on Wagner et al. [31] was conducted. The reactions are described in following equations. The first is a reaction of hydroperoxides with sodium iodide in a presence of acetic acid (1H_2_O_2_:1I_2_) (Equation (2)):

H_2_O_2_ + 2NaI + 2CH_3_COOH → 2CH_3_COONa + I_2_+ 2H_2_O (2)

The next step involves reactions of iodine with thiosulfate to determine the hydroperoxide concentration through a quantification of reduced iodine (1I_2_:2I-) (Equations (3) and (4)):

Na_2_S_2_O_3_ + H_2_O → S_2_O_3_^2−^ + Na^+^ + H^+^ + OH^−^ (3)

2S_2_O_3_^2^^−^ + I_2_ → S_4_O_6_^2^^−^ + 2I (4)

PLA samples were immersed into 50 mL of glacial acetic inside an Erlenmeyer flask. Then, 1 g of sodium iodide was added to the flask. Due to the light sensitivity of sodium iodide, the flask was covered with Al foil. Reactions were carried out in an inert (argon gas) atmosphere and away from light to ensure that only the created hydroperoxides were used to oxidize the iodide. The color of the mixture changed to yellow after the iodide was oxidized to iodine. After reaching the titration threshold, a subsequent titration with sodium thiosulfate (0.0005 M) solution resulted in a colorless solution, and the concentration of hydroperoxides per treated area was calculated.

### 2.7. Surface Morphology Analysis

The surface topography for plasma-treated PLA films was analyzed using a confocal method for optical surface metrology using a Leica DCM8 profilometer (Leica microsystems, Wetzlar, Germany). A high-precision surface profiling optical system was used to measure the influence of polymer concentration on nano-fibre morphology. Images were sizes 160 × 130 μm^2^ using 100× objective lens. The mean of arithmetic height (Sa), which was estimated over the entire measured area, was used to quantify the roughness of the surface.

The surface morphology of the PLA samples was investigated by scanning electron microscopy (SEM). Two-dimensional (2D) images of the examined surfaces were taken using the Nova NanoSEM 450 SEM microscope (FEI, Hillsboro, OR, USA). Thin Au layers a few nanometers thick were sputtered onto PLA samples to obtain high-resolution SEM images and to prevent electron accumulation in the measured substance.

Atomic force microscopy (AFM) was used to examine the surface topography/morphology of PLA samples using an MFP-3D system (Oxford Instruments Asylum research, Abingdon, Oxford, UK) equipped with an AC160TS cantilever with tip (Al reflex coated Veeco model-OLTESPA, Olympus, Tokyo, Japan). Under atmospheric conditions, scanning was conducted using air tapping mode (AC mode) from a surface area of 5 × 5 μm^2^. Alternatively, the value of the roughness parameter (Ra–arithmetic mean height of line) and line profile were evaluated from images collected from the AFM Z-sensor.

### 2.8. Chemical Composition Investigation

Fourier transformed infrared spectroscopy with attenuated accessory (FTIR-ATR) was used to examine the chemical composition changes in the plasma-treated and modified PLA samples. The Spectrum 400 (Perkin Elmer, Waltham, MA, USA) was used to characterize the chemical composition of the PLA samples and identify the functional groups introduced after the plasma treatment and grafting reactions. After background (air) subtraction, all measurements were obtained using eight scans with a resolution of four. Qualitative data were gathered about the absorption of chemical groups in the middle infrared region (4400–500 cm^−1^).

X-ray photoelectron spectroscopy (XPS) was used to quantify the changes in chemical structure caused by plasma treatment and modification of the PLA surface. The device used for this analysis was an AXIS XPS (Kratos Empirical, Manchester, UK). A spherical mirror analyzer and a delay detector are included in the XPS device, which provide good spectral resolution and flexibility for rapid chemical composition screening. Data can be collected at a depth of 1–10 nm using this system.

### 2.9. The Evaluation of Nano-Mechanical Properties

The surface nano-mechanical properties of the PLA samples were determined by a MFP-3D AFM system (Oxford Instruments Asylum research, Abingdon, Oxford, UK) using an amplitude modulation–frequency modulation (AM–FM) mode. The probe was simultaneously excited at its specific resonant frequency and another eigenmode in this approach. The topographical/morphological features of the PLA samples were determined using the fundamental resonance, while the nano-mechanical properties were assessed using the frequency and amplitude shift of other eigenmodes. The interaction stiffness (ΔkFM) was estimated using the frequency shift (Δf) according to the Equation (5):

Δk^FM^ ≈ 2k_c_ × Δf/f_c_ (5)

where kc denotes the cantilever’s spring constant and fc denotes the frequency of the cantilever eigenmode. A general Hertz model describing the contact mechanics between the tip and the analyzed sample was used to calculate the sample’s Young’s modulus. The cantilever elasticity (590.41× 10^3^ Pa/Hz) was first determined using polystyrene standard with a known Young’s modulus (3.3 GPa). The absolute values of Young’s modulus of the analyzed PLA samples were then calculated using this cantilever elasticity.

### 2.10. Peel Test Analysis

A peel test was carried out to analyze the adhesion characteristics of the PLA/Al adhesion joints under a 90° peeling angle of the PLA foil from the Al foil. Peeling resistance (peeling force per width) was obtained by these measurements. The tests were performed by means of a Lloyd LS 1K Plus (LLOYD Instruments Ltd., Bognor Regis, UK) friction/peel tester machine. The sample width and length were 2 × 10 cm, and the sample was under peeling for 360 s at a peeling rate of 10 mm/min. To obtain average values of the peeling resistance, five separate readings were carried out.

## 3. Results

### 3.1. Surface Wettability Analysis

**Figure 1:**
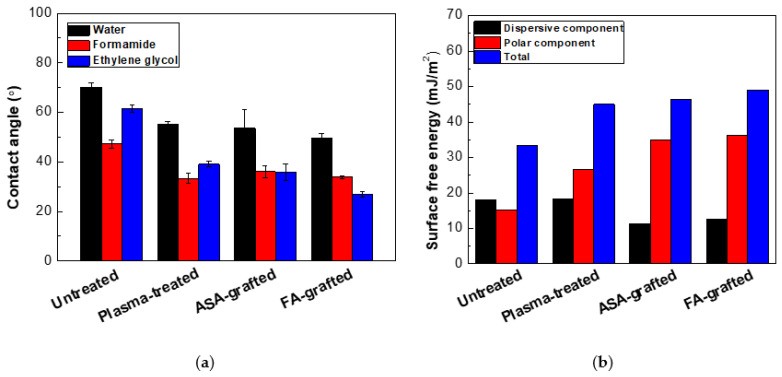
(**a**) The contact angles of the testing liquids on the PLA samples, (**b**) The surface free energy of the PLA samples.

The ability of a liquid surface to bind to a solid surface is characterized by surface free energy and wettability. It means that the lower the contact angle of a sample, the more wettable it is. The effect of RF plasma treatment on the wettability changes of prepared PLA films was investigated using contact angle measurements. The changes in the surface wettability of the modified samples are summarized in Figure 1 and Table 1. Surface-free energy and its components were determined using water (relatively high surface tension ~72.1 mN/m, polar component = 52.2 mN/m and disperse component = 19.9 mN/m) [41], formamide (surface free tension = 56.9 mN/m, polar component = 33.4 mN/m, and dispersive component = 23.5 mN/m) [40], ethylene glycol (surface free tension = 48.0 mN/m, polar component = 19.0 mN/m, and dispersive component = 29.0 mN/m) [42]. For the untreated PLA, the contact angles obtained with the three test liquids: water, formamide, and glycerol were 70.2°, 61.5°, and 47.2°, respectively. A hydrophobic surface is defined by contact angles greater than 90 degrees [39]. As a result, the untreated PLA surface has a hydrophilic behavior with a low total surface free energy (33.3 mJ/m^2^), and a relatively high polar component (15.2 mJ/m^2^). The polar functional groups present on the surface increase surface wettability. Therefore, it was observed that 30 s of RF the plasma treatment of PLA films led to the improvement of wettability, confirmed by the decrease of contact angles as result of the incorporation of new polar functionalities (mainly containing oxygen), etching and ablation processes. The addition of new oxygen-containing functional groups resulted in a 45.0 mJ/m^2^ and 26.8 mJ/m^2^ increase in total surface free energy and polar components, respectively, while the contact angle of water was reduced to 55.1°. Plasma treatment modified only the topmost layers (tens nm) of the material, leaving the bulk of the material unchanged. In order to improve wettability and therefore adhesion characteristics, grafting of the PLA surface by antibacterial agents was performed. The grafting of the polymer surface by polar compounds enhanced the polarity due to the defined structure attachment [24]. Therefore, ASA and FA acid were grafted on the surface of the prepared PLA film a using low-temperature plasma acting as an initiator for radical grafting reactions. The effect of ASA or FA grafting on the PLA surface was demonstrated in terms of its surface wettability and adhesion properties. The graft yield was 5.3% and 3.6% for the PLA surface modified by ASA and FA, respectively, indicating a multilayer formation of both ASA and FA. Moreover, the total surface free energy increased significantly for the PLA surface modified by ASA and FA to 46.4 mJ/m^2^ and 49.0 mJ/m^2^, respectively, as result of increased polar component (34.9 mJ/m^2^ for ASA and 36.3 mJ/m^2^ for FA). It can be concluded that grafting of FA and ASA resulted in significant improvement to the wettability of the PLA surface represented by the polar component of the surface free energy.

### 3.2. Hydroperoxides Determination

**Figure 2:**
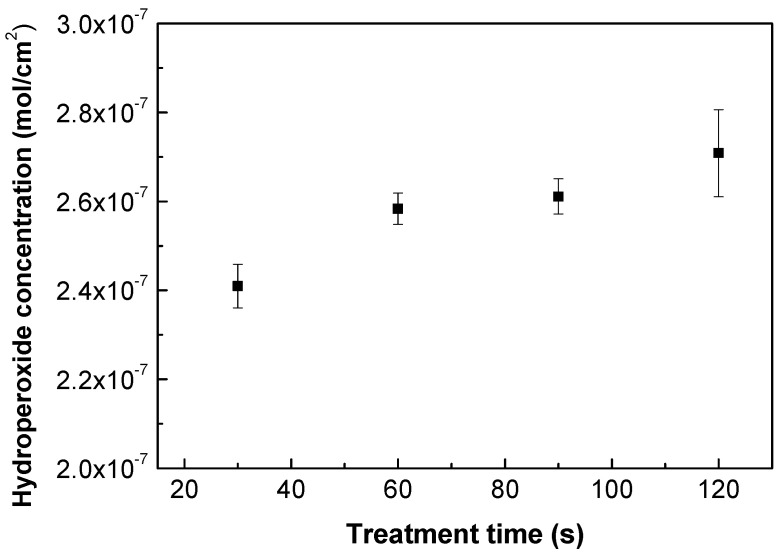
Hydroperoxide concentration vs. treatment time of plasma-treated PLA samples.

As plasma treatment incorporates functionalities on the treated polymer surface, various forms of functional groups can be identified (mostly oxygen-containing). Nonetheless, the majority of free radicals are transformed into peroxides through air exposure before and after plasma treatment [43], and the quantity of peroxide content in either the infrared FTIR or XPS change spectra is difficult to discern. Therefore, an iodometric titration represents a suitable quantification approach according to Wagner et al. [44], which can be used to quantify the concentration of the created hydroperoxides after the plasma treatment process. Grafting reactions by FA or ASA occur with alkoxy radicals, which are created by the decomposition of hydroperoxides [45,46]. Moreover, water-soluble ASA acts as free radical scavenger [47,48] forming ascorbate radicals by electron transfer to alkoxy radicals, and therefore these ascorbate radicals are able to interact with other radicals or unsaturated carbons formed by plasma treatment. Figure 2 depicts the results obtained from the iodometric titration of plasma-treated PLA samples. Plasma treatment took place at different treatment times. The application of iodometric titration indicated that the level of hydroperoxides slightly increased with the time of treatment, rising from 2.4 × 10^−7^ mol/cm^2^ to 2.7 × 10^−7^ mol/cm^2^ for 30 s and 120 s, respectively. This shows that the longer exposure times of the polymer samples do not significantly increase hydroperoxide formation, and thus that the surface is saturated with a certain amount of peroxides at this optimum time [49]. Therefore, 30 s of treatment time was chosen for subsequent grafting reactions and to avoid potential degradation processes, which could occur using longer treatment times.

### 3.3. Chemical Composition Investigation

**Figure 3:**
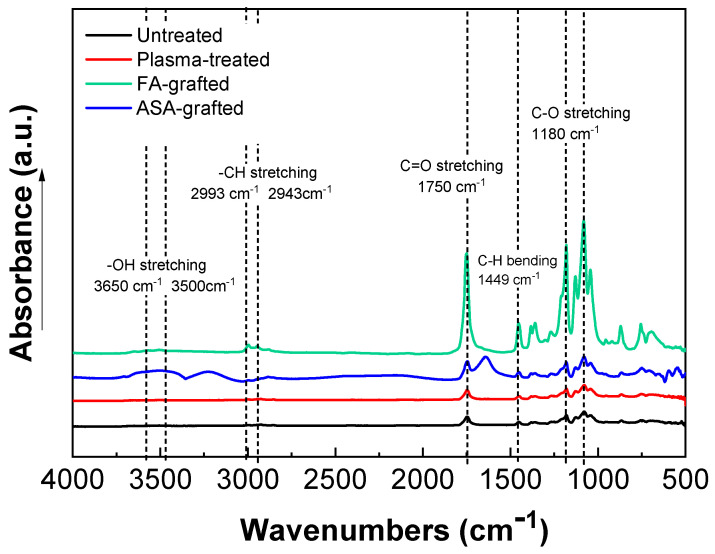
Fourier transform infrared spectroscopy (FTIR) spectra of PLA samples.

The chemical composition of the PLA samples was examined using FTIR-ATR spectroscopy after a modification step and surface oxidation caused by plasma treatment [31]. The data of the FTIR-ATR study show that there are structural changes in the molecular chains of PLA after the RF plasma treatment represented by slight changes in the peak intensities of the groups containing oxygen (Figure 3). The important regions of the PLA absorbance bands in the FTIR-ATR spectrum are at maximum of the wavenumbers 1750 cm^−1^ for the C=O stretching of the ester group, 3650–3000 cm^−1^ for the –OH stretching, 2993 cm^−1^ and 2943 cm^−1^ for –CH stretching, 1450 cm^−1^ for C–H bending, and 1180 cm^−1^ and 1085 cm^−1^ belonging to the C–O. Plasma treatment of PLA film resulted in slightly decreased intensity of –OH (3650 cm^−1^ for free and 3500 cm^−1^ for H bonded bonding) indicating chain scissoring in the PLA structure that resulted in subsequent functionalization by other functional groups such as C=O. Therefore, C=O and C–O (secondary alcohol) increased after plasma treatment. Moreover, in the PLA films modified by ASA and FA, a new absorption band at 1680 cm^−1^ developed that was attributed to C=O stretching of the acid group from the grafted ASA and FA species. These observed changes in the FTIR-ATR spectra of the prepared PLA films proved the functionalization processes occurred during the plasma treatment process. The FTIR spectra of modified PLA films by ASA and FA are illustrated in Figure 3.

**Figure 4:**
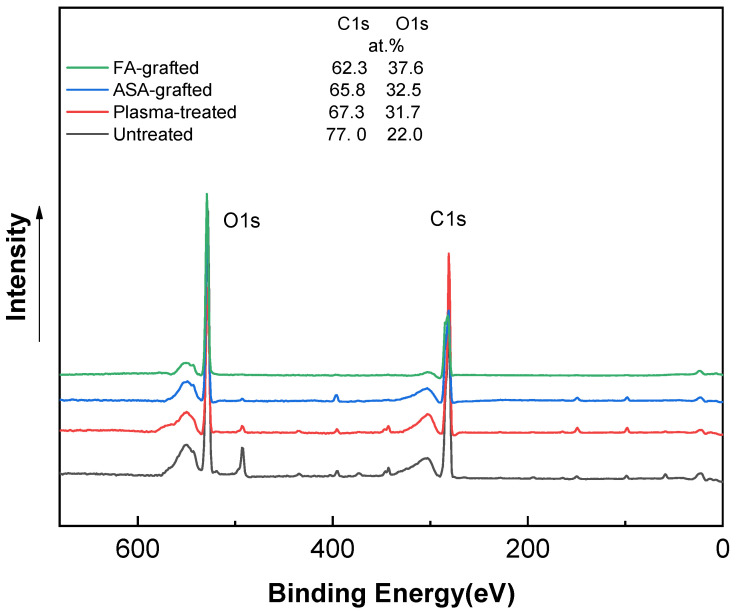
X-ray photoelectron spectroscopy (XPS) spectra of PLA samples.

The XPS technique was used to determine the chemical composition of PLA samples. Figure 4 shows the XPS spectra of the PLA samples after the plasma treatment and modification step. The XPS spectrum for the untreated PLA reveals two main contributions corresponding to C1s at ~285 eV and O1s at ~530 eV, attributable to the PLA’s chemical composition, distinguished by the existence of oxygen in the polymer chain [3,23,37]. The incorporation of new functional groups in the PLA surface was caused by plasma treatment. This functionalization is primary generated by oxygen-rich species because of their high reactivity and, to a lesser extent, nitrogen-rich species [50]. As a result, the peak intensity corresponding to the O1s transition increased significantly, while the peak C1 contribution decreased significantly. The etching process and the formation of radical and oxygen-containing groups led to reduction of the intensity of the C1 peak to 67.3 at.% [23]. The oxygen-containing groups in ASA and FA were primarily responsible for the increase in at.% of the O1 peak caused by the antimicrobial agent grafting on the PLA surface. The O1 peak in the ASA-grafted PLA samples was 32.5 at.%, while the C1 peak intensity was 65.8 at.%. Furthermore, due to probably the presence of nitrogen-containing impurities, the N1 intensity increased slightly to 1.8 at.% when compared to the plasma-treated PLA sample. In contrast, a significant increase in the intensity of the maximum O1 peak, achieving a value of 37.6 at.% indicated the incorporation of the FA grafted to the PLA surface.

### 3.4. Surface Morphology/Topography

**Figure 5:**
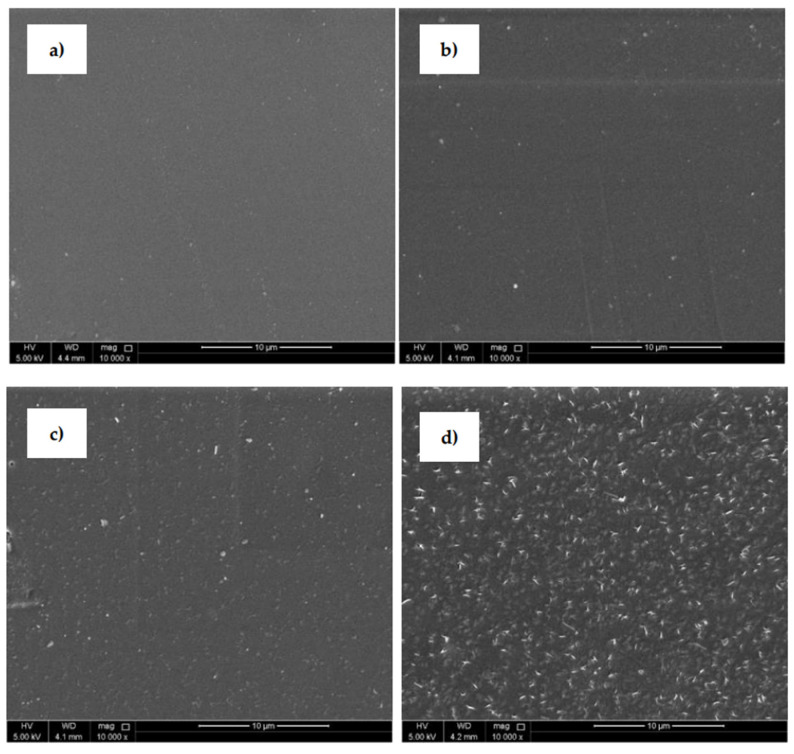
Scanning electron microscopy (SEM), images of PLA: (**a**) untreated, (**b**) plasma-treated, (**c**) ASA-grafted, and (**d**) FA-grafted.

SEM (Figure 5) was uses to analyze the surface morphological changes of the PLA samples after the plasma treatment and modification steps. The SEM image of the untreated PLA (Figure 5a) shows a homogeneous surface with a relatively smooth and uniform appearance. The PLA surface treated with plasma discharge (Figure 5b) reveals an entirely different surface morphology compared to the untreated surface. Small spots, grooves and cavities were observed on the substrate where some removal occurred and some roughness changes were produced. This phenomenon was aided by the physical bombardment of the surface with high-energy particles generated during plasma generation [31,43]. The PLA samples grafted with ASA and FA experienced clear surface morphological changes that demonstrated areas on the surface as grafting occurred. PLA films modified by ASA (Figure 5c) and FA (Figure 5d) were coated by specific layers on the film surface, which confirmed the presence of an antimicrobial agent after the modification process. It can be concluded that modification of PLA by ASA and FA led to an overall roughness increase as the films were coated with antimicrobial agents.

**Figure 6:**
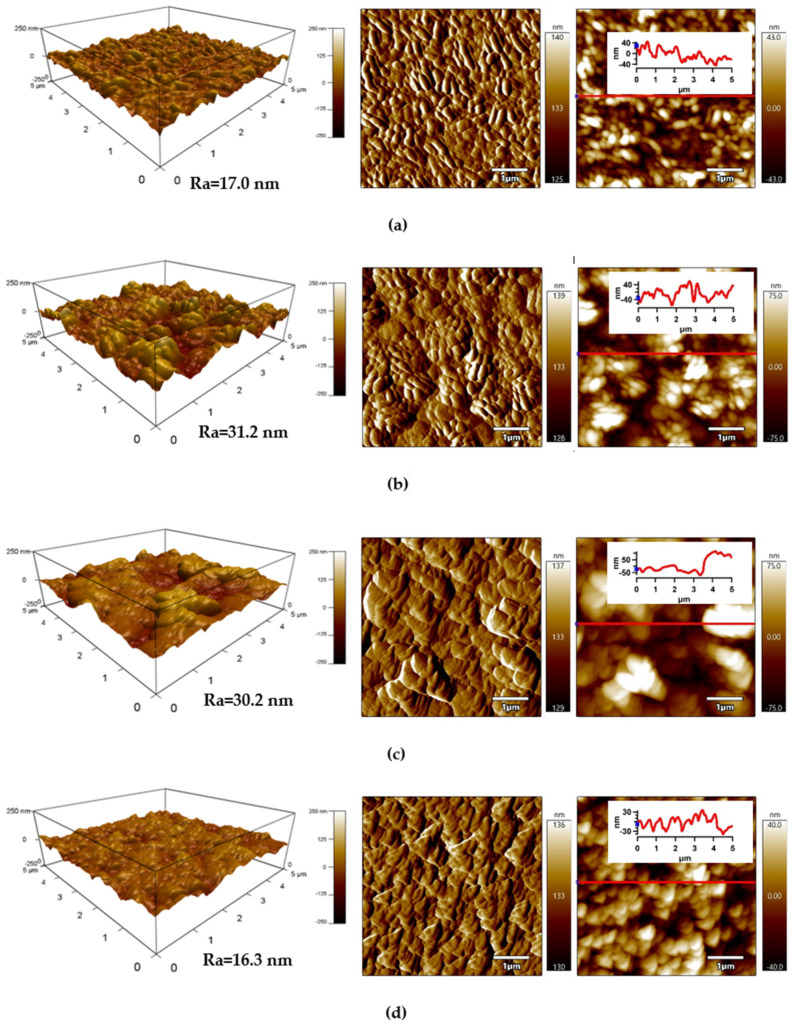
Atomic force microscopy (AFM) images (from left to right: 3D Height, Amplitude, ZSensor including line profile) of PLA: (**a**) untreated, (**b**) plasma-treated, (**c**) ASA-grafted, and (**d**) FA-grafted.

The detailed surface topography and morphology of PLA samples was obtained by AFM from 5 × 5 μm^2^ surface area. The AFM images (Figure 6) provide information about the roughness of the PLA surface after each modification step. The surface of the untreated PLA had a characteristic texture and uniform topography/morphology originating from the preparation process, while Ra (arithmetical mean height of line) was 17.0 nm (Figure 6a). Plasma treatment led to noticeable changes in the surface area of PLA and the surface roughness increased as result of the bombarding action of plasma reactive species with the polymer surface responsible for etching reactions. The Ra parameter of the plasma-treated PLA sample increased to 31.2 nm (Figure 6b). Modification of PLA by ASA and FA led to a formation of specific layers, while Ra was 30.2 nm and 16.3 nm, respectively.

### 3.5. Mechanical Properties

**Figure 7:**
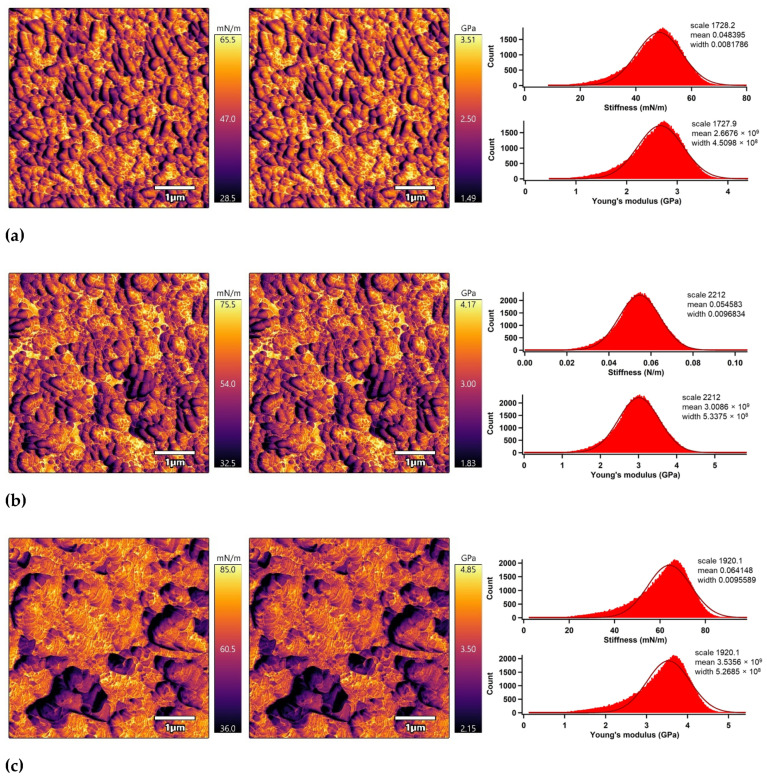

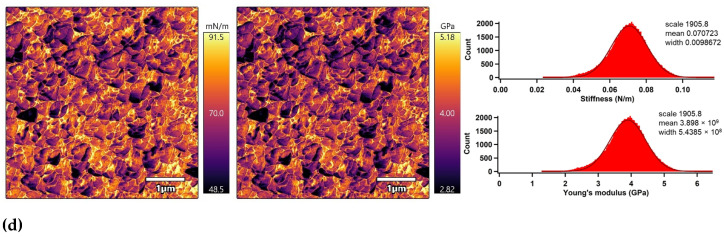
AM–FM AFM images (left: stiffness, right: Young’s modulus) and related histograms of PLA: (**a**) untreated, (**b**) plasma-treated, (**c**) ASA-grafted, and (**d**) FA-grafted.

The nano-mechanical properties of the PLA surfaces were analyzed using an advanced AM–FM mode of AFM in parallel with topography/morphology measurements [50]. The AM–FM AFM images representing the stiffness and Young’s modulus distribution in the entire surface are shown in Figure 7. In addition, a mean value of stiffness and Young’s modulus was calculated as an arithmetic mean value of the measured distribution using Gaussian related histograms [51], which are summarized in Table 2. The untreated PLA surface exceled in uniform distribution of stiffness and Young’s modulus in the entire 5 × 5 µm^2^ surface area. The mean of stiffness and Young’s modulus were 48.4 mN/m and 2.7 GPa, respectively. Plasma treatment was responsible for slightly increasing the nano-mechanical properties of the PLA surface due to structural reorganization caused by ablation, etching, functionalization or crosslinking processes [52], while the stiffness and Young’s modulus increased to 54.6 mN/m and 3.0 GPa. Modification of the PLA samples by ascorbic acid and fumaric acid was responsible for a slight enhancement of the nano-mechanical properties in the surface area due to the formation of compact grafted layers covalently bonded on the PLA surface [53,54]. The stiffness and Young’s modulus increased to 64.1 mN/m and 3.5 GPa for the PLA modified by ASA and to 70.7 mN/m and 3.9 GPa for the PLA modified by FA.

### 3.6. Adhesion Analysis

**Figure 8:**
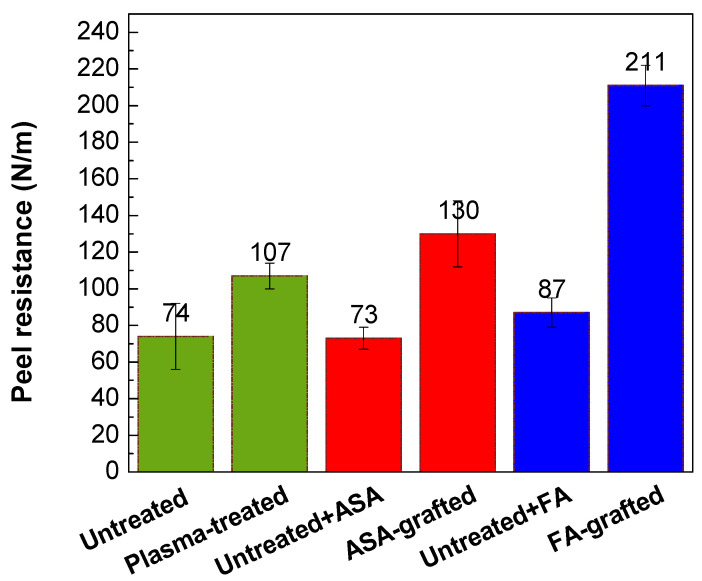
Peel resistance of PLA/Al laminate.

The peel resistance measurements were carried out to evaluate the adhesion properties of the PLA/Al laminate. Higher peel resistance is associated with good adhesion and vice versa, as the result of improved wettability. Figure 8 depicts the changes in the peel resistance of the PLA/Al laminate after plasma treatment and FA and ASA grafting of the PLA surface. Due to the smooth surface and the lowest wettability, the untreated PLA surface had poor adhesion, while peel resistance was 74.4 N/m. On the contrary, the adhesion of the PLA surface was improved after plasma treatment, and peel resistance increased to 107 N/m. This improvement in peel resistance was mainly caused by improvements in wettability and surface roughness, which were due to the incorporation of polar functional groups and the etching reactions in the PLA surface. As illustrated in Figure 8, ASA grafting on the PLA surface resulted in an additional increase in peel resistance (130 N/m) compared with only the plasma-treated PLA samples. The highest wettability was attained by grafting FA onto the PLA surface, the peel resistance increased to 211 N/m (Figure 8). Furthermore, both ASA and FA were subjected to PLA modification without prior plasma pre-treatment (blind test). It was observed that the peel resistance of the untreated PLA modified with ASA and FA had a similar value to that of the untreated PLA sample (73.3 N/m and 87.2 N/m, respectively), indicating that the ASA and FA were not covalently bonded to PLA surface without plasma pre-treatment.

## 4. Conclusions

In summary, the effect of plasma treatment and ASA or FA modification on the surface and adhesion properties of the PLA surface was analyzed by various analytical and microscopic techniques. Due to the incorporation of new polar functional species on the PLA films surfaces, wettability was improved after plasma treatment. Following surface modification of the PLA film with ASA or FA, it was discovered that plasma treatment was successful in grafting ASA and FA on the surface of the PLA film. The plasma treatment and modification of the PLA surface by ASA or FA was proven by various techniques and methods such as profilometry, SEM, AFM, peel tests, contact angle measurements, XPS and FTIR. This modification led to enhanced wettability and therefore improved adhesion characteristics. The peel test of PLA/Al laminate revealed enhancement of the peel resistance of the PLA modified samples, while the peel resistance of PLA modified by ASA and FA increased by 74% and 184%, respectively, compared to untreated PLA. Modification of PLA with plasma technology and subsequent grafting of active species opens an avenue to such tailored properties as biofouling, antibacterial and antiviral activities. It should be pointed out that plasma treatment is not limited to PLA only and can be applied generally to different polymeric surfaces, which makes this approach attractive as well.

## Figures and Tables

**Table 1:** The contact angles, surface free energy and graft yields of the PLA samples.

PLA Samples	Water (◦)	Ethylene Glycol (◦)	Formamide (◦)	Total Surface Free Energy (mJ/m^2^)	Dispersive (mJ/m^2^)	Polar (mJ/m^2^)	GY (%)
Untreated	70.2 (±1.6)	47.2 (±1.7)	61.5 (±1.3)	33.3	18.1	15.2	-
Plasma-treated	55.1 (±1.2)	33.4 (±2.0)	39.0 (±1.2)	45.0	18.3	26.8	-
ASA-grafted	53.7 (±7.5)	36.0 (±3.3)	36.1 (±2.4)	46.4	11.4	34.9	5.3
FA-grafted	49.6 (±1.9)	26.8 (±1.2)	33.8 (±0.4)	49.0	12.6	36.3	3.6

**Table 2:** Nano-mechanical properties of the PLA surfaces.

Sample	Stiffness (mN/m)		Young’s Modulus (GPa)	
Code	Mean	Peak Width	Mean	Peak Width
Untreated	48.4	8.2	2.7	0.5
Plasma-treated	54.6	9.7	3.0	0.5
ASA-grafted	64.1	9.6	3.5	0.5
FA-grafted	70.7	9.9	3.9	0.5

## Data Availability

MDPI Research Data Policies.
